# The Evaluation of Intra-Operative Frozen Section Diagnosis Accuracy of Ovarian Tumors; Old Fashioned Not Out of Fashion

**DOI:** 10.31557/APJCP.2019.20.12.3697

**Published:** 2019

**Authors:** Piyanat Muruthapongsatorn, Narong Inploy, Sinart Prommas, Buppa Smanchat, Kornkarn Bhamarapravatana, Komsun Suwannarurk

**Affiliations:** 1 *Department of Obstetrics and Gynecology, Bhumibol Adulyadej Hospital, Royal Thai Air Force, *; 2 *Gynecologic Oncology Unit, Department of Obstetrics and Gynecology, Faculty of Medicine, Thammasat University, Bangkok, Thailand. *

**Keywords:** Intra, operative frozen section, accuracy, ovarian tumor

## Abstract

**Background:**

The accuracy of intra-operative decision in confirming ovarian tumor malignancy during the operation is vital. Frozen sections are an important intra-operative tool to determine the provisional diagnosis and appropriate treatment of a tumor.

**Study design:**

All records of diagnosed ovarian tumor patients who underwent exploratory laparotomy with intra-operative frozen sections request at Bhumibol Adulyadej Hospital (BAH) between January 2016 and June 2018 were reviewed.

**Main outcome measures:**

Accuracy, sensitivity, specificity, positive predictive value (PPV), and negative predictive value (NPV) of intra-operative frozen and permanent sections were evaluated.

**Result:**

A total of 150 cases were recruited in this study. The mean age was 45.4 years. The number of benign, borderline and malignant ovarian tumors in this study were 97, 9 and 44 cases, respectively. The overall accuracy was 93.3%. Percentage of accuracy, sensitivity, specificity, PPV and NPV for benign, borderline and malignancy were 98.0/94.7/94.0, 100/88.9/79.6, 94.3/95.0/100, 97.0/55.3/100 and 100/99.3/92.2, respectively.

**Conclusion:**

The frozen section results yielded accurate diagnosis for rapid intraoperative evaluation of ovarian tumors. Its high accuracy allows for an appropriate surgical plan to be made in a timely manner. Large sizes and suspected mucinous borderline ovarian tumors reduced accuracy factors of frozen sections.

## Introduction

Ovarian cancer is the second most common female genital tract cancer. The incidence of ovarian cancer was estimated at 6.2 per 100,000 women per year (Global cancer observatory, 2018). Peak incidence was found in post-menopausal women between 56 to 60 years of age (Berek et al., 2019).

Most patients came to the physician and were diagnosed with ovarian masses. Management of ovarian masses depended on the possibility of cancer. There were numerous tests for pre and perioperative differential diagnosis. To date, there are no effective methods for predicting malignancy as ovarian cancer. Serum cancer antigen 125 (CA 125) was a widely used biomarker for ovarian cancer prediction and post-operative follow up (Sölétormos et al., 2016). However, low sensitivity and low specificity for detecting ovarian malignancy were disadvantages of CA 125.

Ultrasonography, computed tomography (CT) scan, magnetic resonance imaging (MRI), risk of malignancy index (RMI) and risk of malignancy algorithm (ROMA) were used to assess the likelihood of ovarian cancer (Fischerova and Burgetova, 2014). However, there was no novel technique for an accurate prediction of ovarian cancer in either the pre or peri-operative period. Frozen sections were by far was the most reliable method used for provisional intra-operative diagnosis of ovarian cancer (Berek et al., 2019). Its results inform the surgeon’s decision between a fertility-sparing or an extended surgery.

This investigation examined the diagnosis accuracy, sensitivity, specificity, negative predictive value (NPV), positive predictive value (PPV) of the intraoperative frozen section regarding malignancy status in ovarian tumor operation.

## Materials and Methods

This research was approved by the ethics committee approval of Bhumibol Adulyadej Hospital (BAH) institutional review board (IRB105/61) following Quality and Transparency of Health Research (EQUATOR) network guidelines. The participants were receiving care at BAH, Bangkok, Thailand. This was a retrospective study reviewing the case record of all patients who underwent intraoperative ovarian tumors with frozen section evaluation between January 2016 and June 2018 at BAH. Inclusion criteria were patients with a diagnosis of ovarian tumors by clinical examination or imaging with consultation for intraoperative ovarian tumors frozen section. Exclusion criteria included cases of incomplete medical records. Study data was obtained from the hospital computerized database. Data collected included basic and clinical characteristics.

Gross specimen of ovarian tumor was evaluated during the operation. The unilateral salpingo-oophorectomy was performed and sent to the pathologist without formalin fixing. The intact ovarian tumor was thoroughly examined and opened by the attending pathologist to seek any suspected area of malignancy. The suspected area of the specimen was sampled for a frozen section procedure. The ovarian specimen was frozen by using an optimal cutting temperature machine (OCT) compound under -20ºC. It was cut under a microtome knife, fixed onto glass slides and stained with hematoxylin and eosin (H and E).

The pathologist immediately reviewed the slides and reported to the surgeon in the operating room within 30 minutes. The remaining ovarian specimen was then fixed in formalin and later prepared for standard paraffin-embedded technique. Final histopathology results from permanent H and E slide were later reported as the gold standard diagnosis.

Frozen section reports were classified as benign, borderline or malignant. Its report was compared to the result of a permanent section. In case of discrepancy between the frozen section and permanent H and E slides, the slides were reviewed by the same and another pathologist to confirm the report. Accuracy, sensitivity, specificity, positive predictive value (PPV), negative predictive value (NPV) of ovarian tumors between frozen section result and permanent section were calculated.

*Statistical analysis*

Statistical analyses of demographic data were performed using SPSS 18.0 (SPSS Inc., Chicago, USA.) Continuous variables were expressed as mean and standard deviation (SD). Categorical data was expressed as number and percentage. Categorical variables were compared using the Chi-square test or Fisher’s exact test. A p-value of less than 0.05 was considered statistically significant.

## Results

This study comprised 150 patients diagnosed with ovarian tumors who underwent intraoperative frozen section evaluation during the study period. The patients’ mean age was 45.4 years (range from 16 to 84 years). Two thirds of the participants were multiparity and premenopausal status. The percentage of fewer than five years of oral contraceptive pill users was eighty-five. Ninety-one percent of the participants had no familial history of cancer. One-third of the study population had an education level of a bachelor’s degree or higher. The demographic data of the study is presented in Table 1. The average size of the ovarian mass from this study was approximately 12 cm.

A comparison of histopathological reports between frozen section and standard H and E staining was presented in Table 2. When frozen sections were reported as benign, a majority of them (97/100) had benign reports from permanent section pathology results. Half of the frozen cases reported as borderline malignancy were matched with the permanent section.

Fifteen cases in this study were initially reported as borderline malignancy of ovarian tumors. Half of these cases (8/15) had a permanent section with concordance reports to frozen section reports. Another half of the cases (7/15) had more aggressive permanent section reports than that of the frozen sections. There were 4 cases of mucinous adenocarcinoma and three cases with high grade serous, papillary and endometrioid adenocarcinoma. All seven cases of discrepancy report between frozen and permanent sections presented with unusually large ovarian massses (average 16.8 cm in diameter).

Thirty-nine percent of the cases (39/100) with benign condition from frozen section histopathological reports underwent conservative surgery, namely ovarian cystectomy and salpingo-oophorectomy as presented in Table 3. One ovarian cystectomy case with benign condition from frozen section report had a borderline ovarian tumor in her final histopathology report. The patient received counseling and was presented with either a reoperation or a close follow up. She chose a close to follow up by serial ultrasounds and tumor markers.

Other cases with a frozen pathological report of benign tumor (61/100) underwent definite surgery. Fifty-nine cases had the same final histopathology report. Only two cases reported a malignancy finding in the final pathological report of a stage IA ovarian cancer without the need for adjuvant chemotherapy. Another one case was stage IC ovarian cancer that required adjuvant chemotherapy.

All cases (35/35) with malignancy reports from frozen sections had cancer in their final reports. The detail of accuracy, sensitivity, specificity, positive predictive value, and negative predictive value were calculated in individual groups and are presented in Table 4.

The diagnostic value of frozen section (accuracy, sensitivity, specificity, negative predictive value, positive predictive value) was summarized and presented in Table 5. Our data was compared to previous literature in other countries (Malipatil et al., 2013; Ayhan et al., 2016; Hashmi et al., 2016; Arshad et al., 2018). All data from previous Thai studies (Boriboonhirunsarn et al., 2004; Tangjitgamol et al., 2004; Wootipoom et al., 2006; Wasinghon et al., 2008; Suprasert et al., 2008) were summarized and presented as summary data in Table 5.

## Discussion

Intraoperative malignancy evaluation of ovarian tumor was important. It is used to rule out any unnecessary surgery or suboptimal surgery. The gold standard of malignancy evaluation was H and E staining. However, H and E staining process took approximately ten minutes per slide for evaluation (Jaafar et al., 2006). Frozen section could be used to determine if there was any malignancy or not during operation. When malignancy was reported from frozen sections, the surgeon had two paths of treatment; either a conservative or radical surgical staging, depending on the progression of disease and fertility needs. In the present study, when the frozen section reported malignancy, PPV was 100 percent. The surgeon could do an aggressive surgical staging with an appropriate medical indication. Literature from India, Bangladesh, and Malaysia showed frozen sections reported malignancy with the PPV at nearly 100 percent (Malipatil et al., 2013; Hashmi et al., 2016; Arshad et al., 2018). For this reason, frozen section was widely accepted for the intraoperative malignancy evaluation.

The overall accuracy of frozen section examination from literature ranged from 83.7 to 99 percent (Malipatil et al., 2013; Ayhan et al., 2016; Hashmi et al., 2016; Arshad et al., 2018). 

**Table 1:** Patient’s Demographic Data (Total n = 150)

Characteristic		Final HPE			
		Benign*	Borderline*	Malignant*	p-value
Age (year)**	45.4 ± 14.1	47.2 ± 13.5	45.0 ± 17.7	41.3 ± 14.0	0.06
Parity					0.75
Nulliparous		37 (61.7)	3 (5.0)	20 (33.3)	
Multiparous		60 (66.7)	6 (6.7)	24 (26.7)	
Menopausal status					0.14
Pre		65 (63.7)	4 (3.9)	33 (32.4)	
Post		32 (66.7)	5 (10.4)	11 (22.9)	
OCP (year)					0.66
< 5		79 (62.2)	8 (6.3)	40 (31.5)	
≥ 5		18 (78.3)	1 (4.3)	4 (17.4)	
Menarche (year)**	13.3 ± 1.5	13.4 ± 1.5	14.2 ± 1.4	13.0 ± 1.4	0.07
FHC					0.79
Positive		8 (61.5)	2 (15.4)	3 (23.1)	
Negative		89 (65.0)	7 (5.1)	41 (29.9)	
Education level					0.62
Primary		21 (60.0)	4 (11.4)	10 (28.6)	
Secondary		44 (67.7)	3 (4.6)	18 (27.7)	
Bachelor or more		32 (64.0)	2 (4.0)	16 (32.0)	

HPE, (Final Histopathological Examination); FHC, (Family history of cancer: colon, endometrium, ovary, breast); OCP, (Oral contraceptive pill); * n (%), ** mean±standard deviation (SD)

**Table 2:** Correlation between Intraoperative Frozen Sections with Final Histopathological Examination (HPE)

	Final HPE			
Frozen	Benign	Borderline	Malignant	Total (n)
Benign	97	1	2	100
Borderline	0	8	7	15
Malignant	0	0	35	35
Total (n)	97	9	44	150

**Table 3:** Type of Operation Decision Based on Frozen Section Histopathology

	Benign*	Borderline*	Malignant*
Type			
Cystectomy or USO	39 (39.0)	0 (0.0)	2 (5.7)
TAH & BSO	61 (61.0)	15 (100.0)	33 (94.3)

*, n (%); USO, unilateral salpingo-oophorectomy; TAH, total abdominal hysterectomy; BSO, bilateral salpingo-oophorectomy

**Table 4:** Diagnostic Value of Intraoperative Frozen Section for Benign, Borderline and Malignant Lesions

	Frozen section		
	Benign	Borderline	Malignant
Accuracy	98.0%	94.7%	94.0 %
Sensitivity	100.0%	88.9%	79.6 %
Specificity	94.3%	95.0%	100.0 %
PPV	97.0%	55.3%	100.0 %
NPV	100.0%	99.3%	92.2 %

PPV, positive predictive value; NPV, negative predictive value

**Table 5:** Accuracy and Sensitivity of Frozen Section for Ovarian Tumor from Selected Studies

	Malipatil	Ayhan	Hashmi	Arshad	Present	Summary data
Number	218	684	141	92	150	1197
Year	2013	2016	2016	2018	2019	2004-2019
Country	India	Turkey	Bangladesh	Malaysia	Thailand	Thailand
Malignant (%)						
Accuracy	96.3	97.4	99.3	91.3	94.0	94.2
Sensitivity	84.9	91.4	96.4	69.2	79.6	85.0
Specificity	100.0	99.7	100.0	100.0	100.0	98.8
PPV	100.0	99.4	100.0	100.0	100.0	97.1
NPV	95.4	96.6	99.1	89.2	92.2	92.9
Borderline (%)						
Accuracy	96.3	97.4	99.6	91.3	94.7	91.7
Sensitivity	86.7	91.9	88.3	76.2	88.9	63.7
Specificity	97.0	97.9	99.3	88.7	95.0	95.0
PPV	68.4	81.4	83.3	66.7	55.3	59.4
NPV	99.0	99.1	99.3	92.7	99.3	95.8
Benign (%)						
Accuracy	97.2	97.7	99.3	85.9	98.0	94.4
Sensitivity	99.3	99.0	100.0	95.6	100.0	98.1
Specificity	92.6	95.3	97.1	85.1	94.3	89.7
PPV	96.8	97.2	99.1	86.0	97.0	92.4
NPV	98.4	98.4	100	95.2	100.0	97.3

PPV, positive predictive value; NPV, negative predictive value

**Figure 1:**
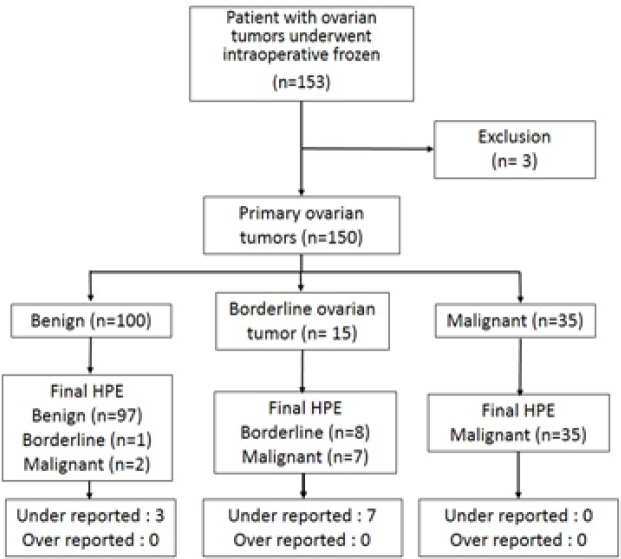
Data Collection of Frozen Section and Permanent Histopathological Report

Borderline ovarian tumor was the most confusing category in diagnosis and treatment. Even though the permanent H and E staining reported borderline ovarian tumor, the pathologist must examine numerous slides carefully to ensure that there was no silent malignancy.

During the operation, each frozen section slide required at least 10 minutes of evaluation time. The operation must be held back for the frozen section report. More frozen slides led to more wasted time for the surgeons. Quality of frozen section staining differed from standard staining. Most pathologists were reluctant to give a borderline ovarian tumor diagnosis from frozen sections. In the present study, there were 15 cases of borderline ovarian tumors from frozen sections. Half of them (7/15) had final pathology reports as malignant and the rest gave the same report as that of the frozen sections. When borderline ovarian tumors were reported from frozen sections, the PPV was around 50 percent. The other studies (Arshad et al., 2018) showed the same result. In this situation, the surgeon must decide to perform an operation either for benign or malignant cases.

Compared to the Ayhan study in Turkey, his study was conducted using a large sample size (687) (Ayhan et al., 2016). PPV of his study was equal to the PPV of our study when the frozen section results reported benign tumors. Reports from India, Bangladesh, and Malaysia showed similar PPV from frozen sections with suspected benign condition (Malipatil et al., 2013; Hashmi et al., 2016; Arshad et al., 2018). In the current study, there were 100 benign ovarian tumor cases from the frozen section reports. Three cases were reported as borderline malignancy from frozen section. All three cases underwent complete surgical staging by the attending physician intraoperative theater.

There was only one case of borderline ovarian tumor preceding by the benign condition from frozen section result. After surgery and permanent section results confirmed, our physicians counseled the patient to either to repeat the operation or return for a close follow up. The patient chose a close follow up method. The other two of them were finally reported as malignant after permanent section performing. The first case, a 21 year old woman with a 20 cm. Ovarian tumor had an intraoperative diagnosis of a benign condition. The surgeon chose to do a conservative surgical staging (unilateral salpingo-oophorectomy, ipsilateral pelvic lymph node sampling, infracolic omentectomy and randomize peritoneal biopsy). Her final diagnosis was ovarian cancer IA (mucinous adenocarcinoma). There was no chemotherapy administration. The second case; 52 years old with a history of hysterectomy underwent complete surgical staging by intraoperative attending physician decision. After surgery, adjuvant chemotherapy was administered.

The limitations of the frozen section method were limit slides numbers, poor sample quality, poor quality of staining, and limited time to report (Jaafar et al., 2006).

In previous studies, there were attempts to investigate factors that led to misdiagnosis in ovarian tumor patients. False-negative reports of frozen sections came from: large tumor size, mucinous type, and borderline ovarian pathology. High precaution should be applied in cases with these conditions (Morton et al., 2017; Ayhan et al., 2016; Ureyen et al., 2014; Subbian et al., 2013; Boriboonhirunsarn et al., 2004).

Frozen section could not yield an accurate diagnosis in borderline ovarian tumors (Bozdag et al., 2016). Increased numbers of frozen sections could not increase the accuracy of the reading (Ayhan et al., 2016). Prolonged wait times for intraoperative report led to higher costs with patients spending more time under anesthesia and higher incidence of complication from prolonged operative time.

Meta-analysis from Chrocane review in 2016 enrolled 11,181 cases who underwent surgery with frozen section examination (Ratnavelu et al., 2016). One-third of cases (3,200/11,181) were cancerous. In the scenario that borderline was classified benign or malignancy, the false-negative rate was three-times higher at 0.009 (29/3,200) and 0.003 (10/3,200) percent respectively. Previous and current frozen section studies in Thailand were categorized into three tiers (benign, borderline and malignant). The subanalysis contained 1,197 cases. The sensitivity, specificity, PPV, NPV were similar to Ratnavelu’s (Ratnavelu et al., 2016). This was supported by Ratnavelu’s work.

In conclusion, the frozen section examination is an excellent intraoperative evaluation tool to determine tumor malignancy. Using frozen section results could reduce unnecessary surgery when the frozen section reports a benign condition. When the report was either borderline or malignant, the result prevented the suboptimal surgical removal. The large ovarian tumor and suspected mucinous tumor could reduce the accuracy of frozen section readings. The surgeon should gather the other clinical information to decide on appropriate surgery when faced with this condition.
